# Transition in morphology and properties in bottom-up HPHT nanodiamonds synthesized from chloroadamantane

**DOI:** 10.1039/d4na00802b

**Published:** 2025-03-10

**Authors:** Stepan Stehlik, Petr Belsky, Tomas Kovarik, Zuzana Nemeckova, Jiri Henych, Egor Ukraintsev, Ales Vlk, Martin Ledinsky, Evgeny Ekimov

**Affiliations:** a Institute of Physics of the Czech Academy of Sciences Prague Czech Republic stehlik@fzu.cz; b New Technologies – Research Centre, University of West Bohemia in Pilsen Pilsen Czech Republic; c Department of Material Science and Metallurgy, Faculty of Mechanical Engineering, University of West Bohemia Pilsen Czech Republic; d Institute of Inorganic Chemistry of the Czech Academy of Sciences Husinec-Řež Czech Republic; e Faculty of Environment, Jan Evangelista Purkyně University in Ústí nad Labem Ústí nad Labem Czech Republic; f Faculty of Electrical Engineering, Czech Technical University in Prague Prague Czech Republic; g Vereshchagin Institute for High Pressure Physics, Russian Academy of Sciences Troitsk Russia

## Abstract

Direct bottom-up high pressure high temperature (BU_HPHT) synthesis of nanodiamonds (NDs) from organic precursors excels in the ability to control the size of the resulting BU_HPHT NDs *via* the temperature of the synthesis. Here we investigated size-dependent thermal, colloidal, and structural properties of the BU_HPHT NDs and focused on the transition in morphology and properties occurring at around 900 °C (≈2 nm). Using transmission electron microscopy, small angle X-ray scattering and atomic force microscopy we show that the sub-900 °C samples (<2 nm NDs) do not have nanoparticle character but 2D platelet morphology with sub-nm unit thickness. Correspondingly, sub-900 °C samples (<2 nm NDs) have a negative zeta potential and hydrophobic character and should be considered as a form of a molecular diamond. The above-900C (>2 nm NDs) samples have nanocrystalline character, positive zeta potential and are dispersible in water similarly to other types of hydrogenated NDs. By *in situ* Raman spectroscopy experiments, we show that the transition is also related to the structural instability of the oxidized sub-2 nm BU_HPHT NDs.

## Abstract 2

Nanodiamonds synthesized by the bottom-up method under high pressure and high temperature (BU_HPHT) show a transition in properties and morphology at approximately 2 nm.

## Graphical abstract

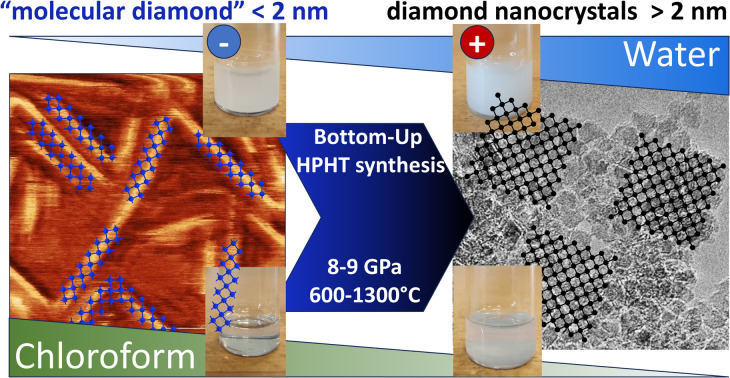

## Introduction

Diamond nanoparticles, so-called nanodiamonds (NDs), are receiving increasing attention due to their unique bulk and surface properties, which provide them with broad application potential in diverse fields such as biology,^1^ bio-sensing,^2^ energy storage,^3–5^ photocatalysis^6,7^ or filtration.^8,9^ The fabrication of nanodiamonds with well-defined properties such as size, structure and surface chemistry is still challenging because none of the commercially used ND fabrication techniques can meet all three parameters simultaneously. For example, detonation nanodiamond (DND) meets a relatively narrow size distribution, but its structure is highly defective and polycrystalline.^10,11^ On the other hand, single-crystal high-pressure high-temperature (HPHT) NDs, prepared by milling, have a well-defined, monocrystalline structure but do not meet the criteria of narrow size distribution.^12^ These two ND types also have different properties after the same hydrogenation treatment, which does not provide the same surface chemistry, hydration, and electrical properties.^13^ Recently developed bottom-up synthesis at high pressure and temperature (BU_HPHT) from molecular precursors such as halogenated adamantanes is an innovative technique that has been shown to be capable of synthesizing tens of mg of ND in a single cycle and can meet all three of the above parameters simultaneously.^14^ The BU_HPHT technique provides fully hydrogenated single crystal NDs and allows excellent control over the size of the diamond nanocrystals in the sub-10 nm region by varying synthetic conditions such as temperature (600–1300 °C) while maintaining constant time (*e.g.* 2 min) and pressure (8–9 GPa).^15,16^ In addition, the method enables boron^17^ and silicon^18^ doping which may further broaden the ND functionalities toward applications in catalysis, bioimaging or cancer treatment. Recent studies of BU_HPHT of NDs synthesized from chloroadamantane have indicated a transition in morphological, optical^16^ and electrical^13^ properties at approximately 2–3 nm. Specifically, the disappearance of the IR transmission window at 1330 cm^−1^ was accompanied by a transition from the conducting (≈10^−5^ S cm^−1^) to the non-conducting (≈10^−11^ S cm^−1^) state between 2–4 nm. Still, all the samples in the 1.2–8.0 nm size range exhibited a diamond crystalline structure. Based on a thorough experimental and theoretical X-ray diffraction analysis of sub-2 nm BU_HPHT NDs, Stelmakh *et al.*^19^ proposed that the sub-2 nm BU_HPHT NDs synthesized at temperatures below 1300 K are plate-shape grains terminated by (111)B surfaces with three dangling bonds and composed from only six hexagonal carbon layers. Thickness of such plates was suggested to be ≈0.55 nm which raises a question of a kinetic stability considering the previous experimental studies, showing that the crystalline structure of the oxidized nanodiamond is kinetically stable down to 1–2 nm but not below.^20–22^ It remains unclear whether this “stability limit” also applies to hydrogenated nanodiamonds and how this transition manifests itself in properties other than optical and electrical properties. For example, hydrogenated NDs are known to form stable colloidal solutions with highly positive zeta potentials.^23–27^ Formation of the positive zeta potential is associated with a decreased energy barrier for electrons characteristic for hydrogenated diamond surface^28^ leading to a high charge density localized on the DND “non-diamond” surface.^13^ However, no size-dependence evolution of this unique property has been reported.

In this work, we build on previous studies and deeper investigate the discontinuity in the physicochemical properties of BU_HPHT NDs, which appears in the region 2–3 nm. We attempted to answer the question of whether and how the discontinuity manifests itself in colloidal and thermal properties of BU_HPHT NDs. We bring the necessary experimental support for the proposed platelet morphology^19^ and diamond-like molecular structure of the sub-2 nm BU_HPHT NDs that appear to have an atomic structure close to that of the diamond but lack some distinct ND properties such as the resolvable nanocrystals in TEM or the ability to form stable colloidal solutions in water. By *in situ* Raman spectroscopy we finally investigate the nanodiamond structural stability as function of size and surface chemistry.

## Materials and methods

### Materials

The synthesis of the BU_HPHT NDs has been described previously in detail.^15,16^ This study involved the samples synthesized at 600 °C (2 min, 1.2 nm), 700 °C (2 min, 1.3 nm), 800 °C (2 min, 1.4 nm), 900 °C (2 min, 2 nm), 1000 °C (2 min, 2.6 nm), 1200 °C, (2 min, 3.4 nm) and 1200 °C (5 min, 4.6 nm). The obtained powder samples have hydrogen termination^15^ and were used as synthesized without further thermal or chemical treatment. The size values were obtained from Williamson–Hall and Scherrer (for the 111 X-ray diffraction peak) methods for calculation of the crystallite dimensions as described in ref. 15. Despite the simplicity of the method, reasonable agreement with other methods was demonstrated, especially for NDs larger than 2 nm.^29^ Whenever appropriate, we note also the synthesis temperature and time.

DND powder was purchased from New Metals and Chemicals, Japan. To unify the surface chemistry with the hydrogenated BU_HPHT NDs, 400 mg of DND were first oxidized by annealing in air at 450 °C for 30 min and then hydrogenated at 700 °C for 6 hours in hydrogen atmosphere.^30^ DND was used as a referential nanodiamond material only in thermal analysis. DND nanoparticle character as well as colloidal properties are well known and therefore, we did not use DND in other analyses. We also did not use DND as a reference in the *in situ* oxidation Raman spectroscopy analysis because DND remained stable in the used temperature range^31^ and a possible change of their surface chemistry would not be detected properly by Raman spectroscopy.^32^

For some analyses, colloidal dispersions were prepared from BU_HPHT ND samples. The powders were dispersed in a demineralized water (conductivity was 0.5 μS cm^−1^) prepared by reverse osmosis and ion exchange station (Resta, s.r.o.) by means of a rod sonicator (Hielscher UP200 St). The sonication lasted 1 h using 0.5 s on/off cycle. The same sonication protocol was also used to prepare dispersions of selected samples (1.2 nm, 2.6 nm, and 4.6 nm) at a concentration of 1 mg ml^−1^ in chloroform.

### Methods

Simultaneous thermal analysis (TA) was carried out using a TA Instruments SDT 650 simultaneous dynamic scanning calorimetry/thermogravimetric analyzer (DSC/TGA). TA analysis was performed under a flow of synthetic air (sample purge flow: air 100 ml min^−1^, balances: N_2_ 10 ml min^−1^). The ND samples were heated to 700 °C at a heating rate of 10 °C min^−1^ in a platinum crucible.

SAXS measurements were carried out on a SAXSess instrument (Anton Paar) equipped with microfocus X-ray source and single-bounce X-ray optics (Xenocs). The ND powder samples were glued between two pieces of scotch tape and SAXS patterns were recorded in the transmission mode with an exposure time of 10 min using a rectangular imaging plate (storage phosphor screen) as detector. The read-out of the imaging plate was performed on a Cyclone® Plus Storage Phosphor System (PerkinElmer). The obtained 2D scattering patterns were first azimuthally averaged using the software supplied with the instrument, resulting in 1D scattering profiles. The 1D profiles were further processed with Irena software package,^33^ including subtraction of background scattering of the scotch tape and logarithmic data reduction. The final profiles were fitted with models using the Modeling and Unified Fit tools in Irena software.

The morphology of the samples was characterized by transmission electron microscopy (TEM) Talos F200X (FEI). Samples in TEM were inspected on a carbon-coated copper grid which was shortly immersed in ND water colloids prior to the observation.

Dynamic light scattering (DLS) and zeta potential measurements were performed on Zetasizer Nano ZS (Malvern Panalytical) equipped with a helium–neon laser (633 nm). The scattering angle was 173°. The refractive index of bulk diamond (2.4) and the viscosity of pure water (0.89004 mPa s at 25 °C) were used. Every sample was measured three times, and each of the three DLS size measurement consisted of 10 runs lasting 10 s. DLS data are plotted using the *Z*-average value, which is a characteristic value of size, obtained as the intensity weighted mean hydrodynamic size. A mean zeta potential value was calculated automatically in the software from Henry equation after estimation of the electrophoretic mobilities of the particles under the applied voltage in the zeta potential measurement cell. The zeta potential value was calculated as the average from 3 zeta potential values. Both measurements were done in a disposable zeta potential measurement cell with a sample concentration of 0.5–1 mg ml^−1^.

For atomic force microscopy (AFM) analysis, 1 mg of ND powder (700 °C) was dispersed in 1 ml of chloroform (p.a.) using the above-mentioned sonication protocol. For the AFM analysis, the sample was diluted to a concentration of *c* = 10^−3^ g l^−1^ and dropcasted (20 μl) on freshly cleaved HOPG surface. The evaporation of chloroform occurred within few seconds leaving a deposit. The deposit was studied on Dimension ICON AFM (Bruker) in PFQNM (peak force quantitative nanomechanical mapping) mode using SNL-A cantilever (Bruker). Several 100 × 100 nm^2^ and 1 × 1 μm^2^ images were obtained in different parts of the deposit area. Images were obtained at 512 × 512 px^2^ and 1024 × 1024 px^2^ scan sizes, force threshold 1 nN, gain 0.5–1, scanning speed 0.1 Hz.

The Raman spectra were acquired using an InVia confocal spectrometer (Renishaw) equipped with a 442 nm Kimmon Dual Wavelength HeCd laser, model IK5651R-G. For the measurements, the Leica 100× objective (numerical aperture NA = 0.90), dielectric rejection filter, and 2400 grooves per mm diffraction grating were used. The Raman spectra were calibrated on the 1332 cm^−1^ diamond Raman peak. The excitation used for measurement was either 4 mW μm^−2^ or 1.4 mW μm^−2^. The temperature dependence of the PL was measured using a temperature control stage (Linkam). The Raman measurement was carried out on the as received powder samples immobilized on a Si substrate.

## Results and discussion

### Thermal properties of properties of BU_HPHT NDs

**Fig. 1:**
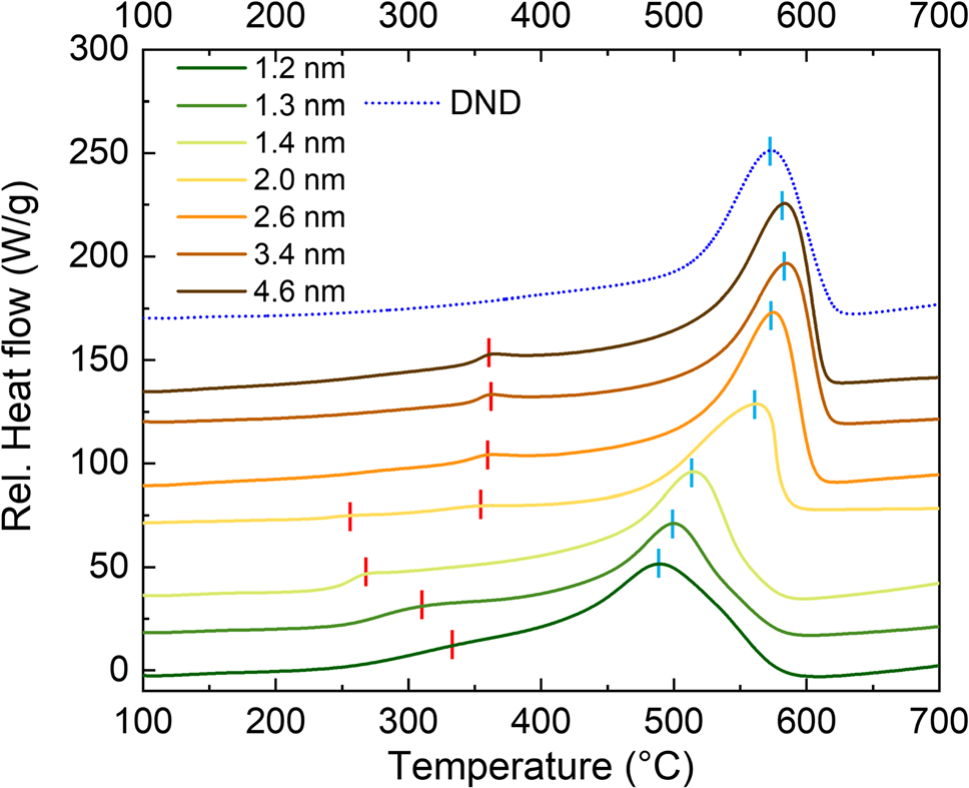
DSC data of the ND samples as a function of ND size. DSC trace of DND sample (dotted blue line) is shown for comparison. The peaks related to the oxidation of C–H_*x*_ bonds and of the combustion are highlighted by short red and pale blue lines, respectively.

The samples were analyzed by thermal analysis to determine C–H_*x*_ bonds oxidation and ND combustion temperatures in dependence on the ND size. Fig. 1 shows the DSC traces of all the studied samples, including hydrogenated DND sample^34^ used here as a reference (blue dotted line). DSC traces of >2 nm samples are very similar, having mainly a smaller exothermic peak around 350 °C, highlighted by a short red line, which we assign to the oxidation of surface C–H_*x*_ bonds, followed by a dominant exothermic effect peaking above 500 °C which originates from the oxidation of the diamond phase, *i.e.*, the ND combustion. The DSC traces of <2 nm ND samples are significantly different from the >2 nm samples as well as from each other. Surprisingly, we observed a decrease in the C–H_*x*_ oxidation temperatures and increase of the combustion temperature with increasing ND size from 1.2 nm to 2 nm. It is obvious that the 2 nm ND sample represents a transient state between the two distinct size regions as it contains characteristics of both <2 nm and >2 nm groups of samples, particularly two C–H_*x*_ oxidation peaks and a broadened and highly asymmetric combustion peak. The DND reference sample showed a broad and indistinct C–H_*x*_ oxidation peak in the 300–500 °C range followed by a clear peak assigned to combustion of the diamond phase.

**Fig. 2:**
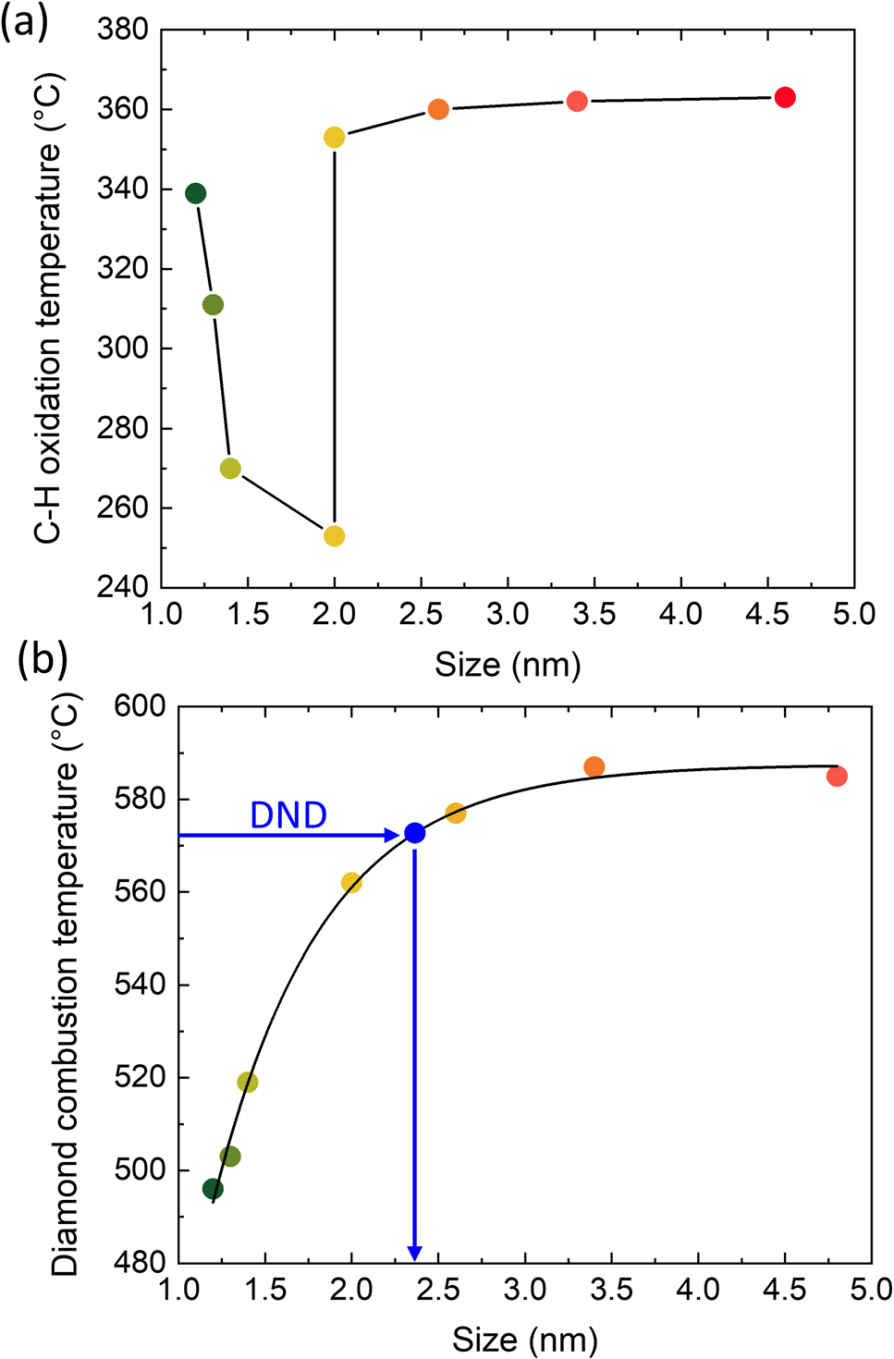
C–H_*x*_ oxidation (a) and ND combustion temperatures (b) as a function of size. The line in (a) is to guide the eye. The data in (b) were fitted by an asymptotic function. The point of DND was placed on the curve taking the DND oxidation temperature to derive a hypothetic ND size that would correspond to “monocrystalline DND”.

Fig. 2 shows the temperatures of C–H_*x*_ oxidation (Fig. 2a) and ND combustion (Fig. 2b) plotted as a dependence on the ND size. The data were derived from the DSC traces (Fig. 1), taking maxima of the relevant peaks. First, the oxidation temperature of C–H_*x*_ decreases with increasing ND size up to 2 nm. Then, there is a step increase and the oxidation temperature of C–H_*x*_ becomes almost constant for ND samples of 2.6, 3.4 and 4.6 nm. This anomalous behavior proves a transition in the surface C–H_*x*_ bond arrangement occurring around 2 nm possibly due to a morphological change as shown below. The ND combustion temperature asymptotically increases with the increasing ND size as highlighted by a good accordance of the asymptotic fit with the data points. The point related to DND was placed on the curve using the temperature of the DND oxidation determined from the DSC trace (Fig. 1). Interestingly, such derived size for DND corresponds approximately to 2.4 nm monocrystalline NDs which is lower than the typical DND size (4–6 nm) and it is most probably associated with the polycrystalline and defective structure of the DND.^11^

### SAXS and TEM analysis

**Fig. 3:**
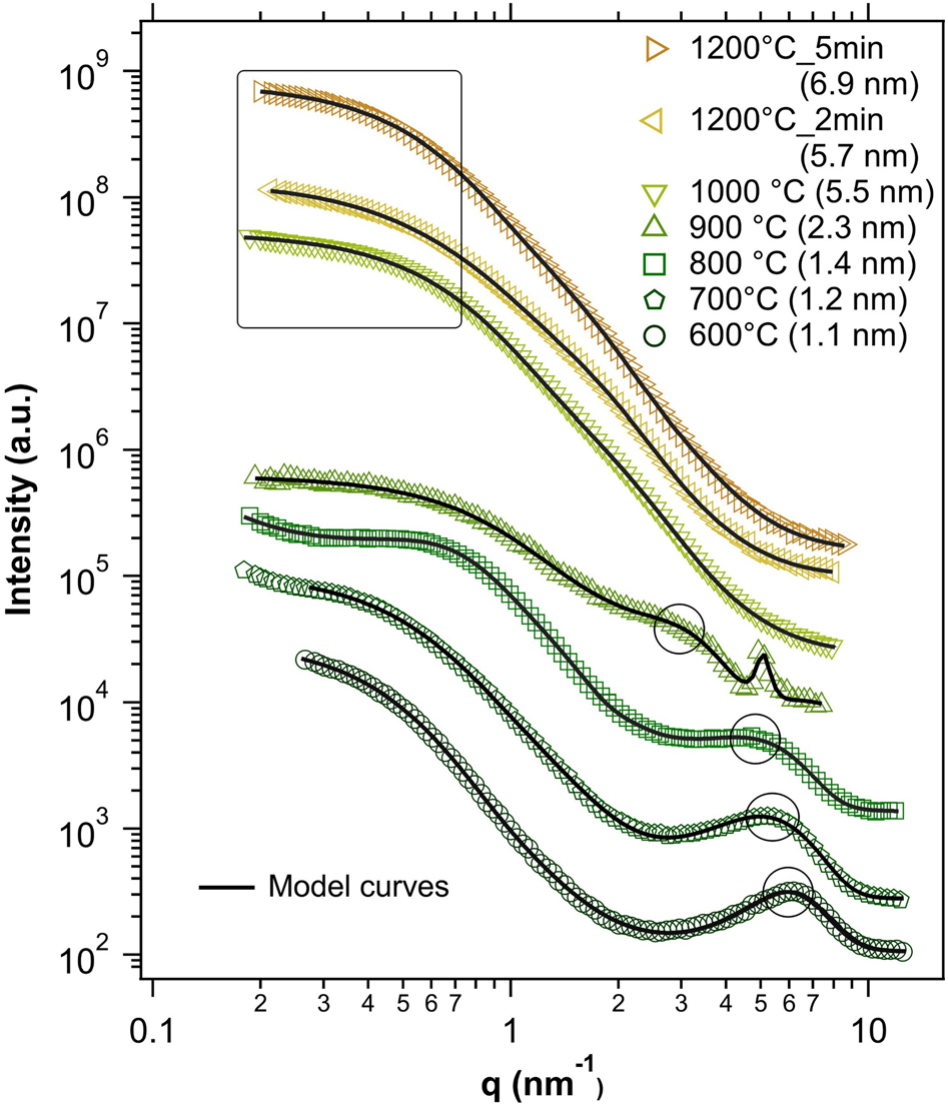
SAXS profiles together with the fitted models; the parts of the curve from which the size of the ND particles was derived are indicated. The derived SAXS sizes are provided in the legend.

The SAXS profiles of all the investigated ND samples are shown in Fig. 3. The SAXS data of the 1.2–2 nm ND samples (600 °C to 900 °C) clearly show two features: a knee on the left side of the profile and a peak on the right side of the profile. The peak on the right side indicates a periodic structure on the nanometer scale with a period around 1 nm (the peak) while the knee on the left indicates objects with size of a few nanometers. Model fitting in *Irena* software (modeling tool) was applied on these profiles using a diffraction peak for the right peak and a unified level^35^ for the left knee. The diffraction peak provided the Bragg distance (period) equal to 1.1 nm, 1.2 nm and 1.4 nm for samples synthesized at 600 °C, 700 °C and 800 °C, respectively. These values match well to the XRD-derived size values.^15^ The double of the gyration radius (2*R*_g_) corresponding to the left knee for 600 °C, 700 °C and 800 °C resulted to 8.0 nm, 6.7 nm and 4.5 nm, respectively. Thus, with increasing ND synthesis temperature, the Bragg period of nanostructures increased whereas the size corresponding to the left Guinier knee decreased (the right peak shifted to the left and the left Guinier knee shifted to the right, *i.e.*, towards each other). The shift of Guinier knee to smaller sizes with increasing synthetic temperature from 600 °C to 800 °C correlates with the anomalous evolution of the C–H_*x*_ oxidation temperature shown in Fig. 2a and is associated with a gradual morphological change. The profile at 900 °C was evaluated in the same manner, *i.e.*, as a diffraction peak and a Guinier knee – in this case they overlap. The corresponding dimensions were 2.3 nm (diffraction peak) and 3.9 nm (2*R*_g_ pertaining to the Guinier knee). Besides, in this sample also a sharp SAXS peak (crystalline reflection) was observed at ∼0.5 nm^−1^ corresponding to a Bragg distance of 1.2 nm.

The particle sizes of samples synthesized at a temperature >900 °C based on the fits (taken as 2*R*_g_ of the left Guinier knee) were: 5.5, 5.7, and 6.9 nm for 1000 °C, 1200 °C 2 min and 1200 °C 5 min, respectively. These values are somewhat higher than the XRD-derived size values. To describe the ND size with one simple number for each sample, the *d*-spacing pertaining the diffraction peak (Bragg distance) was taken for samples 600–900 °C and 2*R*_g_ obtained from fitting the Guinier knee for the remaining samples (1000–1200 °C). ND sizes obtained in this way are summarized in Fig. 3.

**Fig. 4:**
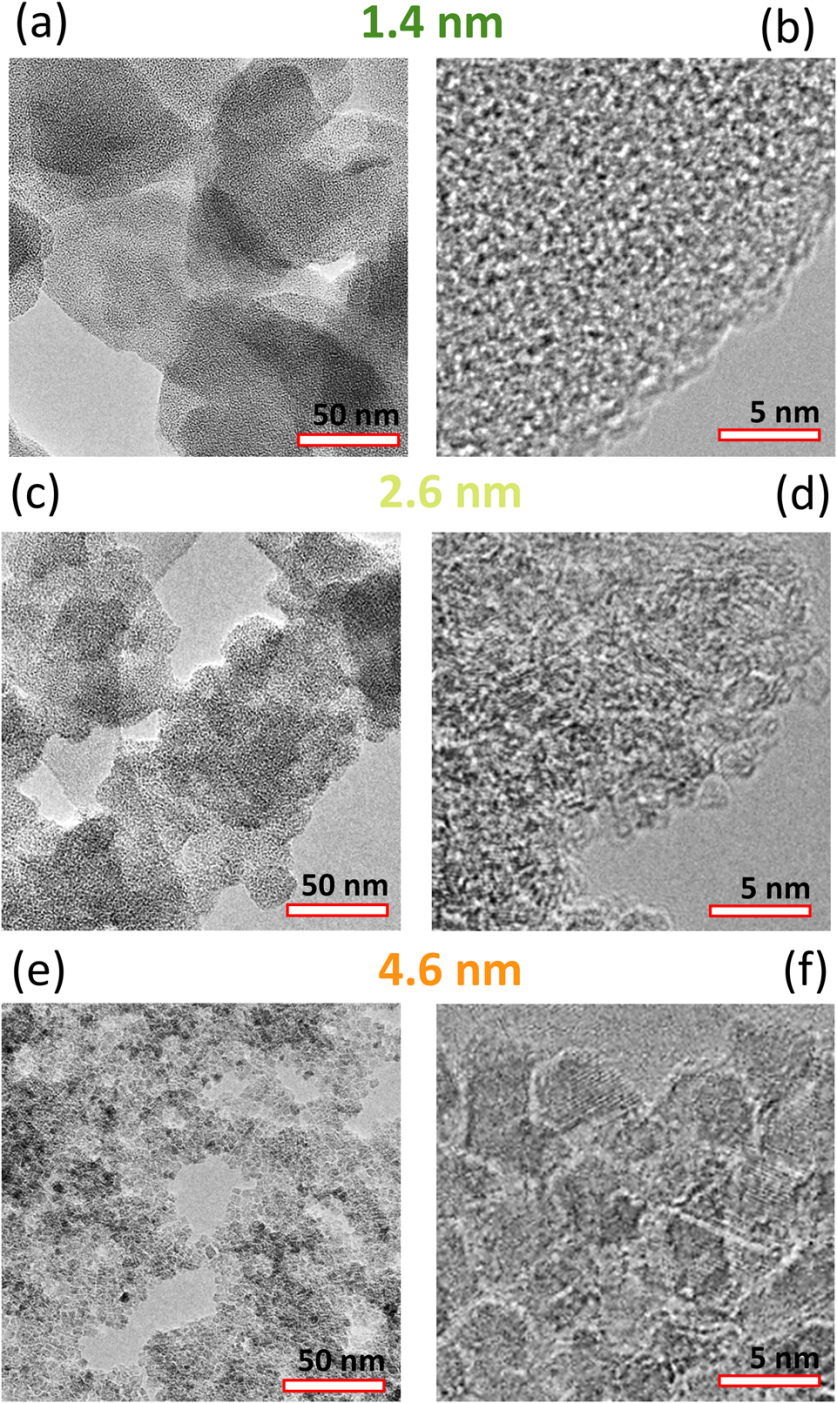
Lower and higher magnification TEM images of the 1.3 nm (a and b), 2.6 nm (c and d), and 4.6 nm (e and f) samples.

The transition of the ND morphology around 2 nm was visually documented by TEM. We have analyzed the three representative samples; below the transition (1.3 nm), just above the transition (2.6 nm), and far above the transition (4.6 nm). The 1.3 nm sample is characteristic by a 2D-like morphology of the individual objects laying over each other on the TEM grid (Fig. 4a). Importantly no individual nanocrystals can be resolved even at a higher magnification (Fig. 4b). The results show that the sub-2 nm samples do not have a typical nanoparticle character although structurally they do consist of a diamond structure down to 1.2 nm as demonstrated by their characteristic Raman spectra and XRD patterns.^15,29^ The nanoparticle character starts to appear in the 2.0 nm sample (not shown) and becomes clearly apparent in the 2.6 nm sample where the 2D-like morphology is still noticeable (Fig. 4c) but the individual NDs can be already recognized at higher magnification (Fig. 4d). The 4.6 nm sample already exhibits a clear nanoparticle character, on both lower (Fig. 4e) and higher (Fig. 4f) magnification.

Overall, the SAXS and TEM data correlate well with the TA data, confirming that a sample synthesized at 900 °C represents a transition between a complex morphology with an ordered superstructure on the nanometer scale (600–800 °C) and well defined, predominantly homogeneous ND particles appearing at temperatures above 900 °C.

### Colloidal properties and zeta potential reversal

**Fig. 5:**
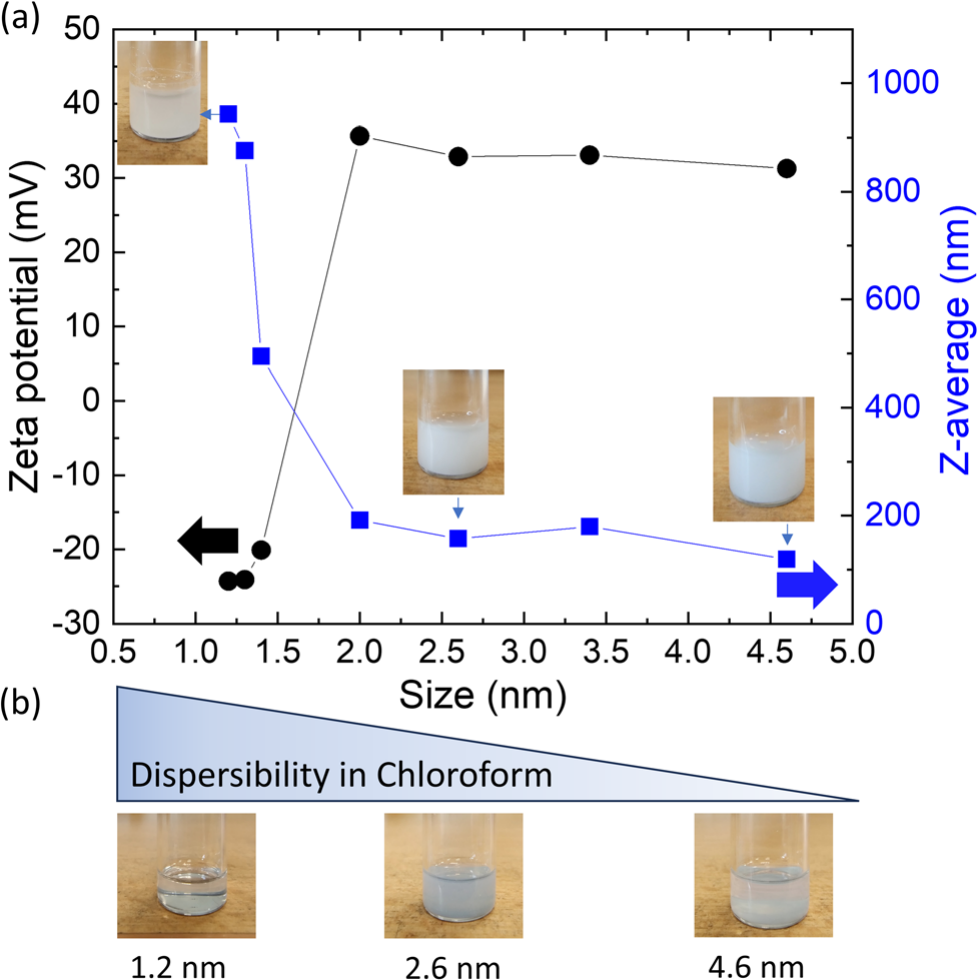
*Z*-Average (blue) and zeta potential (black) of the BU_HPHT NDs as function of size obtained by DLS and zeta potential measurements. Selected samples are accompanied by photographs of their dispersions (a). BU_HPHT NDs dispersions of selected samples in chloroform with an indicated decrease in dispersibility with increasing size (b).

Fig. 5a shows the DLS data plotted as *Z*-average and zeta potential data of the obtained BU_HPHT ND water dispersions. Both DLS and zeta potential data show a clear evolution in the colloid-forming properties of NDs under the identical sonication condition; samples below 2 nm in size have a negative zeta potential, disperse poorly in water, and do not show long-term colloidal stability; a flocculation occurs within a day. As soon as the size exceeds 2 nm, significantly better dispersed colloids with positive zeta potentials were obtained. However, long term stability (for months) exhibited only the 4.6 nm sample, while the samples 2.6 nm and 3.4 nm remained stable only for two weeks. Truly single-digit ND dispersion was not achieved in any sample with the applied sonication protocol. The tight interparticle bonding resembles the properties of DND with their strong interparticle bonds that can be broken up only by highly energetic processes like zirconia beads milling^36^ or salt-assisted ultrasonic deaggregation^37^ and thus may be recommended also for the BU_HPHT ND.

Colloidal stability of NDs is related to zeta potential. Hydrogenated NDs are known to have positive zeta potential.^13,23,25^ Here we show that the zeta potential polarity is size-dependent and positive zeta potential and reasonable colloidal stability appears above 2 nm. Below 2 nm BU_HPHT ND have a negative zeta potential and do not form stable colloids in water, *i.e.* they are hydrophobic and reasonably dispersible in organic solvents such as chloroform. Fig. 5 shows photographs of the dispersions of selected samples 1.2 nm, 2.0 nm and 4.6 nm in water (Fig. 5a) and in chloroform (Fig. 5b). It is noticeable that while the sample 1.2 nm is poorly dispersible in water, it is well dispersed in chloroform. In contrast, the sample 4.6 nm makes stable colloid in water but not in chloroform. The 2.0 nm sample represent a transition between these two.

The behavior of the sub-2 nm samples is similar to organic or carbonaceous materials like graphite^38^ or hydrophobic polymers^39^ that were reported to be hydrophobic while having a negative zeta potential. The observed zeta potential reversal in BU_HPHT ND signalizes rather sudden evolution of a property, that is responsible for the repeatedly reported highly positive zeta potential of hydrogenated NDs.^23–27^ A recent study highlighted important role of a specific surface structure and/or structural defects in the formation of the positive zeta potential of the hydrogenated NDs.^13^ Here we clearly prove that this effect is also related to appearance of diamond crystalline structure in the form of ≥2 nm diamond nanoparticles as shown and discussed below.

### AFM analysis of a sub-2 nm sample

**Fig. 6:**
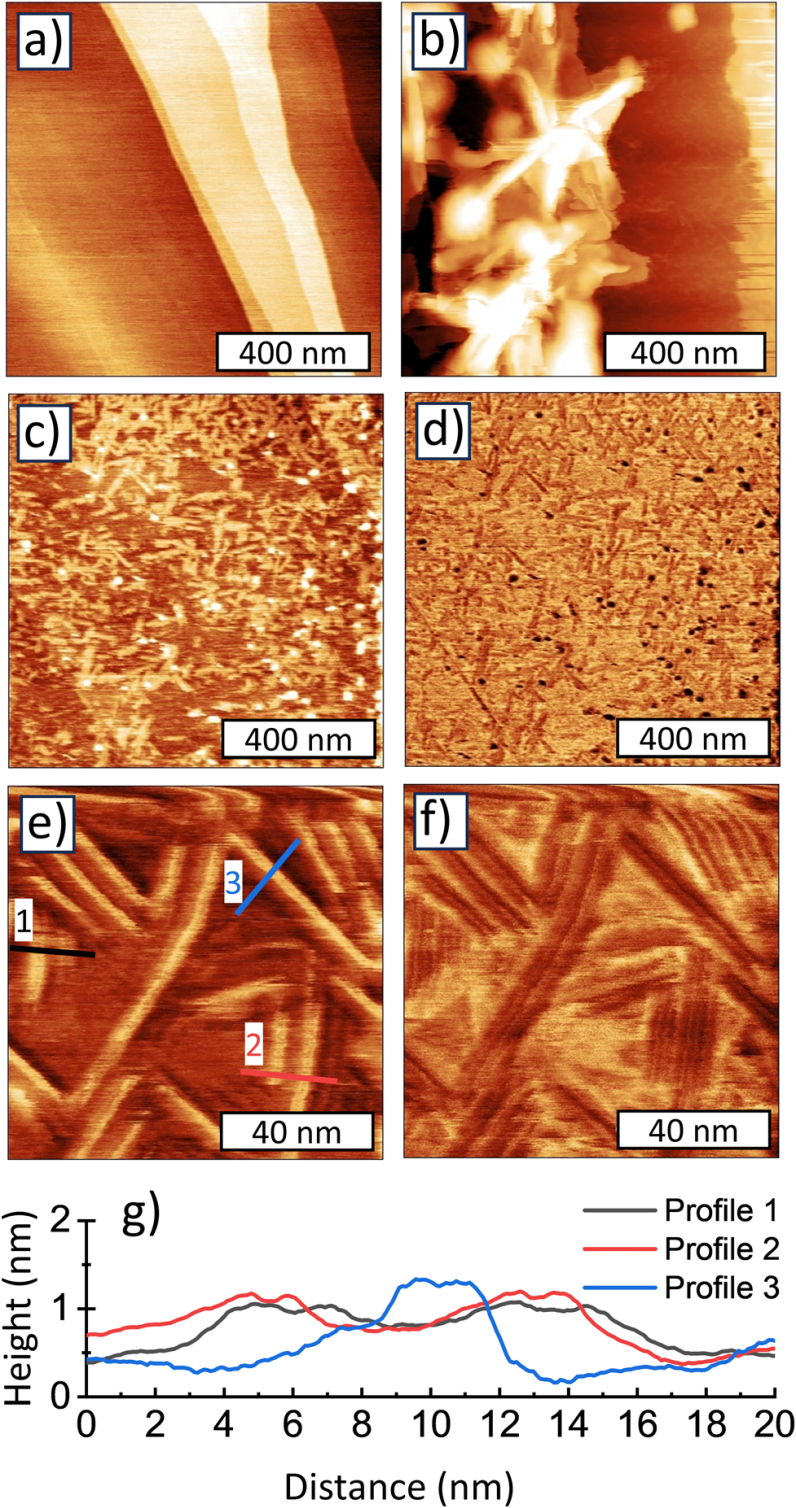
AFM topography of a clean, freshly cleaved HOPG substrate (a). AFM topography (b, c and e) and adhesion (d and f) images of 700 °C sample (1.4 nm) deposited from chloroform solution on freshly cleaved HOPG substrate. *Z* scale = 2 nm (a, c and e), 30 nm (b), 3 nN (d), and 1 nN (f). Three characteristic height profiles from the molecular nanodiamonds are shown in (g).

Good dispersibility of the sub-2 nm samples in chloroform gave us an excellent opportunity to investigate the morphology of a sub-2 nm sample. We used a representative 1.3 nm sample, synthesized at 700 °C, dispersed in chloroform and deposited it on an atomically flat HOPG substrate for a detailed AFM inspection. Fig. 6a shows AFM topography of the clean, freshly cleaved HOPG substrate which is characteristic of atomically smooth areas often used for AFM investigation of various molecules and their self-assembly. Fig. 6b shows a topography of a region close to the center of the deposit, *i.e.* the region with the highest concentration of the material. The topography inspection reveals vertically and horizontally oriented 2D formations, which resemble those observed by TEM in Fig. 5a and most probably correspond to a non-dispersed content. AFM topography (Fig. 6c and e) and adhesion (Fig. 6d and f) of regions several microns away from the droplet centers already show a deposit originating from the well-dispersed chloroform-based colloid. There are two types of objects on these images: long and flat stripes with height of ∼1 nm and length ∼100 nm and larger globular objects with height ∼2 nm and concentration ∼100 objects per μm^2^. In some of the stripes a finer structure can be seen especially in the adhesion channel. We suppose that the stripes consist of fully dispersed “molecular-like” ND whose self-assembly may be guided by HOPG lattice similar to larger molecules such as helicene-based macrocycles adsorbed on HOPG.^40^ We assume that the globular, ∼2 nm objects are not yet dispersed clusters of the molecular-like NDs, therefore not assembling to stripes. Please note, that both globular objects and the stripes have smaller adhesion between the tip apex and the HOPG substrate. In addition, the height profiles plotted in Fig. 6g show that the height of NDs is 0.5–1.2 nm, *i.e.* somewhat smaller than the SAXS-derived size of 1.2 nm.

In the recent report, careful XRD experimental and theoretical analysis of the sub-2 nm ND was performed.^19^ These samples were identical to those studied here, so direct comparison is possible. The authors concluded that the BU_HPHT ND samples with average XRD grain size from 1.2 up to 2.5 nm consists of plates built from only six hexagonal carbon layers having 0.55 nm in thickness.^19^ The TEM investigation of sub-2 nm samples also revealed a 2D morphology of sub-2 nm samples (Fig. 4). Thus, it can be assumed that the different observed sub-1.2 nm AFM sizes (height) may correspond to stacks of such molecular-like NDs platelets. We assume that a direct observation of the individual plates in HRTEM is difficult^16^ because such molecular-like structures may be highly sensitive to electron beam in HRTEM and deteriorate quickly. Nevertheless, less energetic techniques such as XRD or Raman spectroscopy proved its C-sp^3^ structure. Here we showed that such a molecular-like diamond does not have the characteristics for hydrogenated ND such as dispersibility in water associated with the formation of positive zeta potential. Instead, we found that the 1.2 nm NDs were dispersible in chloroform confirming a non-polar character of the constituting units unlike the H-NDs. The formation of such a molecular diamond is specific to the BU_HPHT approach used here, as it has never been reported in other ND synthesis methods such as top-down HPHT or detonation which provide exclusively diamond nanocrystals. Based on the previous works,^41,42^ the BU_HPHT ND synthesis process can be briefly described as follows. With increasing pressure, the distances between chloroadamantane molecules become shorter, initiating polymerization of saturated hydrocarbons that is not typical for normal conditions. The presence of halogen heteroatoms in the molecule makes the intermolecular reaction C_10_H_15_Cl + C_10_ H_15_Cl → C_20_H_30_Cl + HCl even more likely than the C_10_H_16_ + C_10_H_16_ → C_20_H_30_ + H_2_ reaction. The intermolecular interactions preserve the sp^3^ hybridized structure of saturated hydrocarbons, leading to the formation of diamond-like clusters. Nucleation of nanodiamonds takes place at temperatures above 500–600 °C.^14–16^ Temperature plays a crucial role in the local diffusional rearrangement that governs the growth of nanodiamonds, presumably through a recrystallization process above 900 °C.

### *In situ* oxidation and ND stability by Raman spectroscopy

**Fig. 7:**
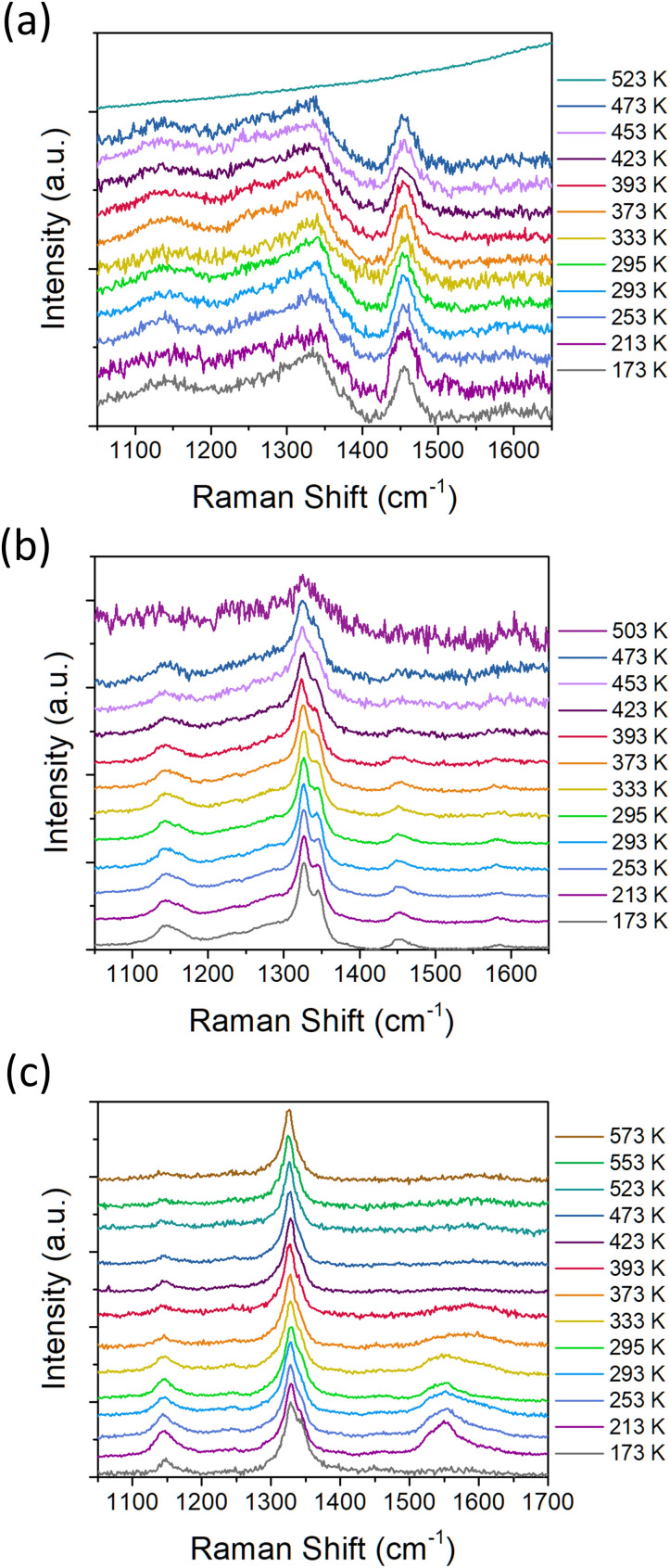
Temperature-dependent Raman spectra of 1.2 nm (a), 2.0 nm (b) and 4.6 nm (c) samples.

We further investigated the oxidation behavior of the three representative samples (1.2 nm, 2.0 nm and 4.6 nm) by *in situ* Raman spectroscopy. The motivation for this analysis was to correlate the thermal processes indicated by the thermal analysis with the structural information obtained by the Raman spectroscopy. Fig. 7 shows the Raman spectra of 1.2 nm (a), 2.0 nm (b) and 4.6 nm (c) samples as dependence on the heating stage temperature on which the samples were placed. The room-temperature Raman spectra of the samples were reported earlier in.^15,29^ Briefly, the Raman spectra contain mainly the peak at ≈1330 cm^−1^ originating from the diamond structure, which is below 2.6 nm significantly broadened and shifted due to the phonon confinement effect.^29,43^ Except the diamond Raman peak, the spectra contain additional features at 1150, 1450 and 1340 cm^−1^ which originate from the bending modes of the surface C–H_*x*_ bonds.^16,29,44^ The feature at 1340 cm^−1^ is overlapped with the diamond Raman peak due to its coupling with the diamond phonon, however, its origin remains unclear and is specific to BU_HPHT NDs.^44^

Another work proposed that the observed two Raman split-bands are due to a plate-like morphology of the diamond grains and may be associated with the inner part and with the edge shell of the plate grains.^19^ Note the absence or very weak signals coming from the sp^2^-C. Such phase and spectral purity as well as the pronounced surface C–H_*x*_ bonds allows for the Raman spectroscopy monitoring of the ND thermal oxidation and its impact on the ND structure. All the measurements started at the temperature of −100 °C and then the temperature was increased step by step until the spectral features of NDs disappeared or the limit of our setup was reached (300 °C). It is worth noting that the provided temperature values correspond to the temperature of the heating/cooling plate and that the absorbed laser radiation could unintentionally increase the sample temperature as well.^45^ This additional heating is highly dependent on the exact measurement position, as it depends on the size and thickness of the illuminated spot and its heat dissipation, *i.e.* level of its contact with the Si substrate. We can approximately estimate this additional heating by comparison of the temperature-dependent Raman spectra with the DSC data depicted in Fig. 2. To minimize the risk of the sample damage by laser heating or any other irreversible photo-induced change, every spectrum was acquired at a new and fresh measurement position. This also helped to verify the homogeneity of the samples, but it introduced an error into the data.

For the smallest NDs of 1.2 nm (Fig. 7a) we were able to perform the spectral acquisition up to 270 °C. Due to relatively low signal, a higher laser power of approximately 4 mW μm^−2^ was needed to obtain clear spectra. The 1.2 nm ND sample shows no significant changes in the Raman spectra up to 200 °C (Fig. 7a). The increase to the final temperature of 250 °C results in an abrupt increase in the PL background, and the ND spectrum is no longer detected. We explain this as an irreversible structural change of the 1.2 nm ND sample due to the heating and/or oxidation. Comparing this result with DSC data (C–H_*x*_ oxidation temperature), we can assume that the laser caused an additional rise in the temperature of approximately 90 °C in this case.

The temperature dependence of the Raman spectra of the 2 nm ND is shown in Fig. 7b. For the larger 2 nm NDs, it was possible to use smaller LP of approximately 1.4 mW μm^−2^ and still obtain sufficiently low-noise ND spectra. The spectral evolution is already different from the 1.2 nm ND sample; a decrease in the C–H_*x*_ bending modes intensity is observed above 200 °C. In the spectrum obtained at 230 °C, significantly broadened diamond Raman peak is still visible, but the C–H_*x*_ bending modes have disappeared which indicates achievement of the stable oxidized 2 nm ND. Comparison with DSC results (the second C–H_*x*_ oxidation temperature at 350 °C) suggests that laser absorption causes additional rise in the temperature of about 120 °C. Similar but significantly more pronounced oxidation effect was observed in the 4.6 nm NDs (Fig. 7c). Here, the increase in temperature above 150 °C is associated with the gradual disappearance of the C–H_*x*_ bending mode signals (1150 and 1340 cm^−1^). The 4.6 nm NDs were able to withstand the temperature of 300 °C while finally providing a clearly detectable diamond peak without any additional C–H_*x*_ bending mode or other signals. In fact, the spectrum contains only the Raman diamond peak. Comparison with C–H_*x*_ oxidation temperature obtained by DSC suggests that laser absorption causes additional rise in the temperature of about 60–80 °C.

### Stability of the diamond structure at the nanoscale

Finally, the stability of the diamond structure at the nanoscale is focused on and discussed. Compared to graphite, diamond is a metastable phase, but the conversion of diamond to graphite does not occur under ambient conditions due to the very large activation energy barrier for the conversion of diamond to graphite; diamond would convert to graphite at temperatures >4500 K. Conversion of diamond to graphite requires breaking numerous C–C single bonds with bond energy of 356 kJ mol^−1^. Therefore, once diamond is formed, it cannot convert back to graphite under normal conditions because activation energy is too high. When moving into the nanoscale or even the molecular realm, size becomes another important factor that may reduce the activation energy of the transformation.^46^

**Fig. 8:**
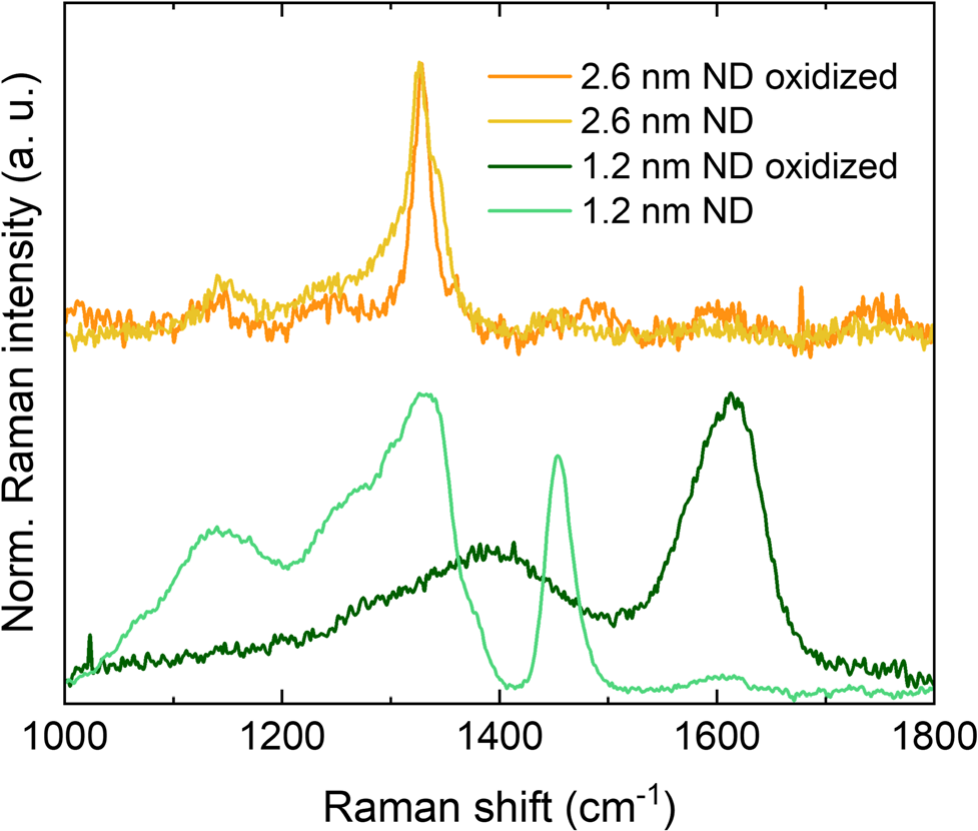
Raman spectra of 1.2 nm and 2.6 nm ND samples in as grown state and after oxidation in the air at 400 °C for 30 min.

To demonstrate the effect of size, we tested the structural (in)stability of the sub-2 nm samples after oxidation and annealed the 1.2 nm and 2.6 nm ND samples in air at 400 °C for 30 min. The temperature was chosen considering the TA data, being above the C–H_*x*_ oxidation temperature and below combustion temperature of both samples (Fig. 2). The Raman spectra of the initial and oxidized 1.2 nm and 2.6 nm samples are shown in Fig. 8. The spectrum of the initial ND sample of 2.6 nm shows a clear characteristic of nanodiamond because it contains a dominant peak at 1328 cm^−1^ related to the crystal structure of diamond. The spectral region 1100–1700 cm^−1^ contains additional peaks around 1150, 1340, and 1450 cm^−1^, which have been assigned to the vibrational modes of surface C–H_*x*_ bonds as mentioned and referenced above. All the features of C–H_*x*_ bending modes disappeared after the oxidation and no sp^2^-C appeared in the 1500–1750 cm^−1^ region. This provides clear evidence that an oxidized diamond crystal structure larger than 2 nm is kinetically stable with a sp^2^-C-free surface, *i.e.*, the surface dangling bonds become saturated with oxygen-rich surface functional groups such as –OH, C=O, and C–O–C,^47^ without surface graphitization. In the 1.2 nm ND sample, the Raman diamond peak is still dominant, but due to smaller size and correspondingly increased surface to volume ratio, the surface C–H bonds provide significantly higher signals. Also, the diamond peak is severely broadened and shifted to lower wavenumbers due to the phonon confinement effect.^48^

After oxidation, the diamond character disappears, as manifested by the appearance of broad D and G bands associated with disordered sp^2^-C, confirming the phase transformation of sub-2 nm ND to an amorphous sp^2^-C carbon structure. This would lead to the conclusion that sub-2 nm oxidized diamond crystalline structure is not kinetically stable. However, it has been predicted theoretically^49^ and demonstrated by STEM, that crystalline structure of the oxidized NDs can be stable down to 1 nm.^20^ It should be noted here that the initial state of sub-2 nm NDs is not nanoparticle-like and the reported size derived from XRD is likely to be an averaged value and does not reflect the high aspect ratio of the possible plate geometry and sub-nm thickness. Our deeper insight provided by TA, SAXS, TEM, AFM and Raman spectroscopy allow us to hypothesize that sub-2 nm ND samples (prepared at temperatures below 900 °C) have a hierarchical morphology on multiple scales with the morphology at the nanoscale corresponding to stacks of constituent sub-nm units that are molecule-like. Since at least one dimension, *i.e.* the thickness of the plate, is less than 1 nm, the oxidation-induced rearrangement of the surface leads to the destruction of the diamond crystal structure and the formation of amorphous carbon.

## Conclusions

Using thermal, microscopic, scattering and spectroscopic techniques, we studied the transition in the morphology and properties of BU_HPHT NDs synthesized directly from chloroadamantane. We have shown that in the 1–2 nm region, the samples, although chemically sp^3^-C, do not exhibit nanoparticle morphology and properties that are characteristic of hydrogenated NDs, such as positive zeta potential and associated colloidal stability in water. Instead, BU_HPHT NDs below 2 nm had negative zeta potential and poor water dispersibility similar to graphite or non-polar polymers. AFM and TEM analysis corroborated the recently proposed platelet-like morphology with sub-nm thickness of the constituting units.^19^ Using Raman spectroscopy, we have shown that BU_HPHT NDs below 2 nm cannot be oxidized without destroying the sp^3^-C structure, which correlates with their sub-nm thickness when considering the theoretically and experimentally proven kinetic stability limit of ≈1 nm for oxidized diamond. Above 2 nm, BU_HPHT NDs acquire a distinct nanoparticle morphology as evidenced by TEM and SAXS, and nanodiamond properties documented by stabilization of their combustion and C–H_*x*_ oxidation temperatures, positive zeta potential and water dispersibility. Above 2 nm, it is also possible to obtain kinetically stable oxidized nanodiamonds without sp^2^-C, as demonstrated by Raman spectroscopy. Therefore, only BU_HPHT NDs larger than 2 nm can be considered as true NDs in terms of nanoparticle morphology and characteristic physicochemical properties. The results presented here fill a gap in the understanding of the stability and properties of ultra-small nanodiamonds and provide important frontiers in applications where sub-5 nm nanodiamonds are considered or required.

## Data availability

The data that support the findings of this study are openly available in Zenodo at http://doi.org/10.5281/zenodo.13844829.

## Author contributions

Conceptualization, S. S., P. B., J. H., A. V., M. L.; investigation, P. B., T. K., E. U., M. N., A. V.; funding acquisition, S. S., J. H., M. L., E. E.; data curation, S. S.; methodology, P. B., E. U., A. V., project administration, S. S.; writing—original draft preparation, S. S.; writing—review and editing, P. B., T. K., J. H., E. U., S. S. All authors have read and agreed to the published version of the manuscript.

## Conflicts of interest

There are no conflicts to declare.
